# Residual Acetabular Dysplasia in Young Children: a Comprehensive Review of Diagnosis and Management

**DOI:** 10.1007/s12178-026-10015-0

**Published:** 2026-02-26

**Authors:** Luke Sang, Nicholas Kelly, Jillian Neuner, Ishaan Swarup

**Affiliations:** Department of Orthopaedics, University of California, San Francisco, USA

**Keywords:** Residual hip dysplasia, Developmental dysplasia of the hip, Abduction bracing, Pelvic osteotomy

## Abstract

**Purpose of Review:**

Residual acetabular dysplasia (RAD) is a common condition where the acetabulum remains shallow or underdeveloped even after treatment for developmental dysplasia of the hip (DDH). Proper management of RAD is crucial in reducing the risk of hip instability and premature arthritis. This review summarizes the most recent evidence related to the diagnosis and management of RAD in patients during their first decade of life, with an emphasis on data that may inform clinical decision-making and highlight areas in need of further investigation.

**Recent Findings:**

Recent studies have shown that rigid abduction bracing significantly improves RAD in patients over six months of age compared to observation only and has minimal treatment complications. Furthermore, bracing has been shown to have a dose-dependent relationship with improvement in acetabular index (AI). Surgical treatment of RAD with pelvic osteotomy significantly improves AI compared to all other treatment approaches, though several studies indicate a reduced effect among older patients. The exact timing and indications for surgical intervention remain controversial.

**Summary:**

The management of RAD remains an active area of research, particularly regarding the timing and indications for surgical intervention. Recent studies have demonstrated the effectiveness of rigid abduction bracing for patients with RAD over the age of six months to assist in acetabular remodeling, with a low complication rate. Pelvic osteotomy provides the most significant improvement in AI compared to all other approaches, with some evidence suggesting that these procedures become less effective as patients get older. However, as the timeline for surgical intervention remains unclear, there is a need for shared decision-making when managing RAD, along with additional long-term prospective studies.

## Introduction

Developmental dysplasia of the hip (DDH) is an orthopaedic condition of infancy and early childhood characterized by abnormal hip joint development, ranging from mild acetabular dysplasia to complete dislocation of the hip[1]. DDH is typically diagnosed through a combination of physical exams, as well as ultrasound or radiographic imaging [2]. DDH can be treated using a variety of methods, ranging from physical therapy, injections, and bracing (e.g., Pavlik harness) to more invasive procedures, such as closed or open reduction or osteotomy [3]. Residual acetabular dysplasia (RAD) in young children is defined as an acetabular deficiency that persists despite successful initial treatment of congenital dislocation in infancy or early childhood [4–6]. While natural childhood acetabular remodeling may lead to the correction of mild DDH, it can also contribute to treatment failure in cases of more severe DDH and thus the development of RAD [7].

### Mechanism and Risk Factors

In RAD, the acetabulum remains shallow and underdeveloped even after intervention. The risk of RAD in children increases with age at initial treatment; patients aged 32 months or older have an estimated 60% chance of developing RAD after treatment [2, 4, 6]. The severity of initial DDH presentation also correlates with an increased risk of developing RAD, as higher pre-operative and post-operative acetabular index (AI) radiographic measurements are associated with greater rates of treatment failure [8].

### Epidemiology

The incidence of RAD varies widely across studies. Following treatment with a Pavlik harness, studies have reported an incidence of RAD ranging from 4 to 30% [9–13]. A meta-analysis by Shaw et al. [14] found an average RAD incidence following Pavlik harness of 9.49% across 17 studies. Meanwhile, in a study examining outcomes of surgical reduction for DDH, it was found that about one-third of patients developed RAD, regardless of the reduction technique used (closed versus open) [4].

### Presentation and Natural Progression

The natural course of RAD varies dramatically and depends on severity. Many mild cases undergo spontaneous resolution due to normal acetabular remodeling following treatment. Imaging is generally used to monitor the progression of RAD over the course of a patient’s early development, and specific measurements, such as the AI, can be utilized to detect instances of failed remodeling. If significant acetabular dysplasia persists at four to five years of age, the literature suggests that secondary intervention is important to mitigate further complications [5, 15]. If left untreated, persistent acetabular dysplasia has a range of complications. Decreased contact between the femoral head and acetabulum leads to increased axial joint load and premature cartilaginous damage [6, 16]. This may result in disability as well as an increased risk of early-onset osteoarthritis necessitating hip preservation surgery or total hip arthroplasty [5, 15, 17].

### Purpose of this Review

Given the potential complications, the purpose of this review is to synthesize current evidence on the diagnosis of RAD to inform clinical decision-making for patients in their first decade of life. The absence of long-term follow-up studies on this topic makes it difficult to identify which children with DDH are most at risk for RAD [18]. Further, the management of RAD remains controversial, with a lack of specific indications for surgical interventions, highlighting the importance of discussing the most updated set of evidence and identifying areas in need of further research.

## Diagnosis

RAD in pediatric patients is most encountered as a continuation or late manifestation of DDH. The clinical presentation of RAD is highly age-dependent and often subtle, particularly in younger children, where skeletal immaturity limits both physical findings and radiographic clarity [19]. In contrast to early presentations of DDH in the neonatal period, residual dysplasia may emerge months to years after treatment or may be incidentally identified in children who were never formally diagnosed but exhibit radiographic signs of abnormal acetabular development [2, 19]. For children approaching school age, symptoms such as gait abnormality, fatigability, or hip discomfort may become more apparent as changes in hip mechanics begin to manifest during walking and physical activity [2]. Early childhood patterns are summarized in Table 1, which outlines common risk factors and clinical findings across the early pediatric years.

**Table 1:** Common RAD risk factors and clinical findings per age groups

Age Group	Medical History / Risk Factors for DDH	Family History of DDH	Physical Exam Findings
< 6 months	Breech presentation, female sex, first-born status, oligohydramnios, swaddling practices	Strong predictive value; screening recommended if positive	May show limited abduction, asymmetric thigh folds, positive Barlow or Ortolani
6–18 months	History of prior DDH treatment, noncompliance with harness, late diagnosis	Still relevant; especially if initial screening was negative	May have persistent limitation in abduction, leg length discrepancy, asymmetric gluteal folds
18 months − 6 years	Delayed walking, history of failed closed reduction, or inadequate follow-up	May influence need for continued surveillance	Subtle Trendelenburg gait, limp, limited internal rotation, often unremarkable if bilateral dysplasia
6–10 years	History of untreated or inadequately treated DDH, residual acetabular dysplasia following early treatment, delayed or missed diagnosis	May support suspicion in cases of delayed presentation or bilateral involvement	May present with activity-related limp, hip or groin pain, positive Trendelenburg sign, limited hip abduction or internal rotation, leg length discrepancy

### Medical History and Risk Factors

Medical history is an essential component of RAD evaluation, as many patients will have identifiable risk factors for DDH that contribute to persistent dysplasia. Breech presentation, particularly frank breech in the third trimester, is the most consistently reported perinatal risk factor [2, 19–21]. Other recognized contributors include female sex, first-born status, oligohydramnios, and postnatal swaddling practices that restrict hip abduction [2, 19–21]. These factors not only support the initial diagnosis of DDH but also inform the likelihood of incomplete acetabular remodeling and the need for extended surveillance. Persistent dysplasia may develop even after early treatment, particularly in patients with breech presentation or older age at initial reduction [19, 20].

### Family History

Family history serves as another important predictor of residual dysplasia. First-degree relatives of individuals with DDH exhibit a significantly elevated risk of acetabular deficiency [20]. This familial predisposition supports the use of extended imaging follow-up in asymptomatic siblings or children with a positive family history, even when early imaging appears reassuring [20].

### Physical Exam

As outlined in Table 1, the Ortolani and Barlow maneuvers are most helpful in diagnosing hip dysplasia in young infants during the first few months of life. The Ortolani test evaluates the reducibility of a dislocated femoral head, while the Barlow test evaluates the potential for dislocation from the acetabulum. These maneuvers are most reliable before the age of three months, after which soft tissue contractures and limited hip mobility reduce their sensitivity and specificity [19]. After this period, the focus of the physical exam shifts to more static findings, including limited abduction of the hip in the supine frog-leg position and leg length discrepancy (notably Galeazzi sign in unilateral cases) [19]. These findings may be subtle and are more easily appreciated in unilateral dysplasia, whereas bilateral involvement often presents with a more symmetric yet globally limited range of motion. As children become ambulatory and especially once they reach school age, gait assessment becomes increasingly relevant because symptoms and functional limitations are more likely to emerge at that stage. A Trendelenburg gait may reflect abductor weakness secondary to mechanical disadvantage from inadequate lateral coverage of the femoral head [19]. In this pattern, during the stance phase on the affected side, the pelvis drops on the contralateral side due to insufficiency of the gluteus medius muscle [19, 20]. Ultimately, the physical examination in RAD is characterized not by dramatic instability but rather by nuanced, often minimal findings. In many cases, a physical exam that appears normal should not delay imaging; plain radiographs remain the most reliable tool for confirming or excluding residual dysplasia in the appropriate clinical context.

### Imaging Modalities and Findings

Radiologic imaging is a key component of both diagnosis and long-term monitoring in RAD. Because clinical examination alone is insufficient for detecting persistent or borderline dysplasia, especially in asymptomatic or bilaterally involved patients, imaging offers objective and reproducible metrics to assess acetabular development, femoral head coverage, and joint congruency [2, 19, 22]. Imaging serves not only a diagnostic role but also guides treatment decisions, tracks remodeling over time, and facilitates surgical planning. The choice of imaging modality, whether radiography, MRI, or CT, depends on the patient’s age, the degree of ossification, and the clinical context [2, 19, 22].

### Radiographs

**Fig. 1:**
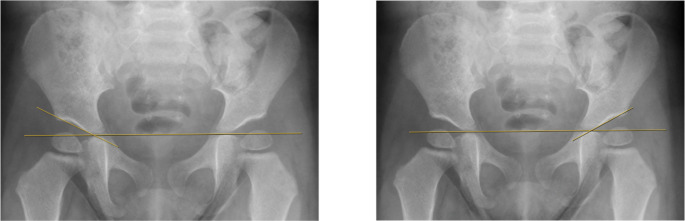
Representative radiographic images for AI in a 2-year-old patient with bilateral acetabular dysplasia. There was an AI of 28 degrees on the left and 30 degrees on the right

Standard anteroposterior (AP) pelvic radiographs remain the primary imaging modality for evaluating acetabular morphology in children over six months of age. Radiographs are widely available, reproducible, and provide reliable bony landmarks once the triradiate cartilage and proximal femoral epiphysis begin to ossify [19, 22]. Among the most commonly used measurements is the AI, defined by the angle between Hilgenreiner’s line and a line drawn along the acetabular roof. AI values decrease with age and are typically below 30 degrees by six months and below 24 degrees by two years. An AI that remains persistently elevated beyond age norms suggests deficient acetabular development and increased risk of degenerative change later in life [2, 19, 22]. An illustration of an example AI measurement is demonstrated in Fig. 1.

Another key metric is the lateral center-edge angle (LCEA), which quantifies the bony coverage of the femoral head by measuring the angle between a vertical line from the femoral head center and a line to the lateral sourcil. An LCEA below 20 degrees is considered dysplastic, 20–25 degrees is borderline, and values above 25 degrees are typically considered normal in children over five years of age [22]. LCEA becomes more reliable as ossification progresses and is best interpreted in children older than five [22].

The Shenton line, a qualitative arc formed by the inferior border of the superior pubic ramus and the inferomedial femoral neck, provides a rapid visual cue for hip alignment. Disruption of this arc suggests femoral head subluxation or superior migration [23]. Additional radiographic indices, such as the extrusion index, can supplement interpretation, particularly in older children and adolescents; however, they are less frequently used in routine early pediatric assessments [22].

### Magnetic Resonance Imaging

Magnetic resonance imaging (MRI) plays an increasingly important role in assessing RAD, particularly in younger children whose hips are largely cartilaginous and are poorly visualized on radiographs. MRI provides unparalleled soft tissue detail, enabling precise evaluation of the cartilaginous acetabulum, femoral head, labrum, and surrounding capsule [2, 24]. Among the MRI-based indices, the cartilaginous acetabular index (CAI) mirrors the AI but uses the cartilaginous acetabular roof as the reference plane. A CAI greater than 10 degrees in early childhood is suggestive of dysplasia [24]. The cartilaginous center-edge angle (CCE), conceptually similar to the LCEA, quantifies cartilaginous femoral head coverage. Values below 23 degrees are consistent with under coverage and may correlate with a future need for surgical correction [2, 24].

### Computed Tomography

Computed tomography (CT), while less frequently used in young children due to radiation exposure, retains a role in select cases, particularly in preoperative planning for complex deformities or in older children nearing skeletal maturity. CT provides detailed osseous visualization and allows for accurate three-dimensional assessment of femoral and acetabular version, as well as for assessing articular cartilage changes [25, 26]. Although MRI is often preferred for its lack of radiation, low-dose CT protocols and 3D reconstructions have made CT increasingly viable when detailed osseous anatomy is required [25, 26].

## Management

The management of RAD remains a challenging issue in pediatric orthopaedics due to differences in progression and overall response to treatment. Furthermore, there is a lack of high-quality data to guide the decision between conservative and surgical treatment options. Based on the current body of evidence, the management of RAD is generally predicated on the patient’s age, with nonoperative approaches such as observation or abduction bracing for younger children and surgical options reserved for older children or those who fail initial treatment [27–30]. Additional considerations for management also include the severity of residual dysplasia and the timing of initial diagnosis. Clinical decision-making centers on enhancing joint alignment and minimizing the long-term risks of degenerative hip disease, while avoiding unnecessary surgical procedures in hips that may naturally remodel over time. The exact timing and specific indications for management decision-making remain controversial, with various studies supporting different approaches as discussed here.

### Nonoperative Management: Observation and Abduction Bracing

For younger children, there is potential for spontaneous resolution of RAD over time without intervention. Thus, an observational approach to managing RAD consists of regular follow-up appointments and imaging. In a prospective longitudinal cohort study by Saeed et al.,[31] the majority of patients with RAD at two years post-bracing treatment for initial DDH had spontaneous resolution by five years (87%). The authors suggested that patients with RAD at two years could be simply followed to determine if surgical intervention is needed at five years [31].

However, other studies have suggested that the natural remodeling of the hip decreases and becomes much less likely as children get older, particularly between the ages of six and 18 months [30, 32]. Rigid abduction orthoses are also often utilized to improve acetabular development in these patients and have been shown to be crucial in various studies [27, 28, 31, 33]. Gans et al. [27] found that patients with RAD at the age of six months who underwent abduction bracing until one year old had significantly better AI compared to those in the observation cohort. Furthermore, Swarup et al.[28] demonstrated a dose-dependent relationship between brace compliance and AI improvement, showing that longer hours of brace wear correlated with greater remodeling. Thus, the use of abduction bracing is generally recommended for patients over the age of six months with RAD to prevent future joint degeneration, specifically given the context of minimal treatment complications. However, while bracing is effective for toddlers, it becomes more difficult and less effective for older patients, around the age of 18 months [34]. Our practice has observed that abduction bracing is less tolerated in these older patients, with consequently decreased compliance. The nonoperative management data discussed are detailed in Table 2.

**Table 2:** Summary of outcomes from recent Conservative management studies in RAD

Study	Cohort	Time Frame	Major Details and Outcomes
Gans et al. [27]	Bracing versus Observation	6 months	A total of 52 patients were included in this study (21 bracing patients and 27 observational patients). Braced cohort had a mean AI reduction of 5.3 degrees, compared to 1.1 degrees in the unbraced group (*p* < 0.001).
Saeed et al. [31]	Post-Bracing for DDH Longitudinal	5 years	181 patients were included in this study. Based on the number of hips, at two years of age, the prevalence of RAD was 43%. This number was reduced to 6% by 5 years. The only predictor of RAD at 5 years was the AI-lateral edge measure.
Swarup et al. [28]	Bracing vs. Historical	6 months	26 patients were included in this study. There was a mean AI improvement of 4.8 degrees. There was a significant correlation between the hours of bracing per day and improvement in AI (*p* < 0.05).
Yilmaz et al.	Bracing in Previous Pavlik for DDH versus No Previous Treatment for DDH	6 months	80 patients were included in this study. Mean AI reduction of 4.9 degrees by end of the study period. There were no differences found in AI outcomes between patients with and without previous Pavlik treatment for DDH (*p* = 0.1).

### Surgical Intervention

**Fig. 2:**
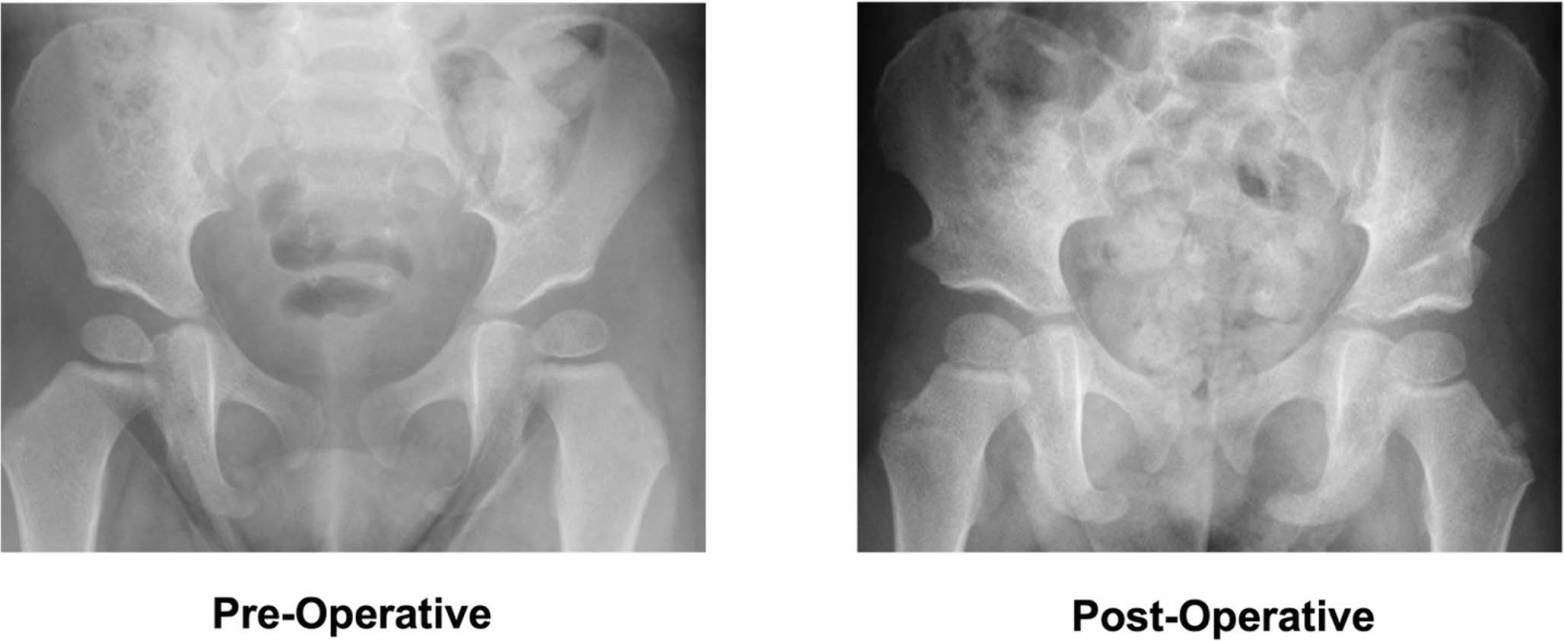
Representative radiographic pre-operative image of a 2-year-old with bilateral hip dysplasia and then imaging post-bilateral Dega osteotomy. Notably, there is a significant improvement in bilateral AI (Left: 28 to 11 degrees, Right: 30 to 15 degrees)

For older children and those with severe RAD or failed nonoperative management, surgical management is often considered. Surgical management typically consists of a pelvic osteotomy with or without a femoral osteotomy. These surgeries are categorized into redirectional (Salter, periacetabular osteotomy, triple pelvic osteotomy), reshaping (Pemberton, San Diego, and Dega), and salvage osteotomies (Chiari and shelf). Redirectional and reshaping osteotomies are the most common and applicable procedures for children in their first decade of life [5]. The specific choice of osteotomy is dictated by patient-specific anatomy, including skeletal maturity, acetabular morphology, and the direction of acetabular deficiency. Reshaping acetabuloplasties are utilized for younger patients with open triradiate cartilage, and are specifically useful for those with a large, shallow acetabulum [35]. Meanwhile, redirectional osteotomies are more commonly utilized in older children, with a triple typically performed in children after the age of six years, where decreasing flexibility of the pubic symphysis limits the corrective capacity of a single-cut innominate osteotomy. Salvage osteotomies are only reserved for patients in whom a concentric reduction is not possible [36, 37]. A pre-operative and post-operative imaging example of a RAD patient receiving bilateral Dega pelvic osteotomy can be seen in Fig. 2.

In this patient population, surgical correction of residual acetabular dysplasia consistently yields significant improvements in AI, often exceeding 18–20 degrees, depending on the technique and patient presentation [2, 38–41]. Aly et al.[38] found an 18.13-degree improvement in AI within weeks following modified triple pelvic osteotomy, while Czubak et al.[40] and Sucato et al.[41] observed similar improvements (19–21 degrees) in longer-term follow-up after Dega and Pemberton/Salter osteotomies. Furthermore, Sucato et al.[41] found that Pemberton osteotomy maintained superior acetabular coverage and joint congruity at skeletal maturity compared to Salter, supporting its long-term durability. These improvements far exceed the AI gains seen with observation or even bracing. Therefore, surgery remains the definitive intervention for restoring hip morphology in cases where nonoperative measures fail or dysplasia persists.

When analyzing the long-term benefits of surgical intervention for RAD, a recent study demonstrated that 90% of surgically treated hips achieved Severin I or II classification, supporting durable anatomic remodeling and long-term joint preservation [42]. This positive effect was more limited in older patients receiving the surgery, which the study deemed to be an age cutoff of 8.6 years [42]. Other studies have also reported durable AI improvements following Dega osteotomy with follow-up duration nearing four years, suggesting that the structural correction achieved is likely to support long-term joint stability [40]. Collectively, these studies reinforce that pelvic osteotomies, when appropriately timed and performed well, not only improve AI but also result in lasting structural correction and favorable radiographic outcomes [38, 41].

However, the benefits of these surgical interventions must be weighed against their potential complications. A systematic review by Tejpal et al.[43] reported a 9.4% complication rate for Salter innominate osteotomies, with avascular necrosis (AVN) as the most common adverse event. Czubak et al.[40] similarly found that three out of 52 hips (5.8%) developed AVN following Dega osteotomy. Aly et al.[38] reported a higher rate of three delayed unions in 15 hips (20%), following modified triple pelvic osteotomy, though no AVN nor neurovascular complications were noted. Thus, the timing and indications that necessitate surgery for RAD are crucial to parse out. This area remains unclear, with some studies suggesting the need for early intervention to maximize remodeling potential [30, 44, 45], while others have pushed for surgical intervention only if the hip has been given a sufficient time for natural resolution [46]. Given the paucity of robust literature regarding this topic, decision-making for surgery is often made based on clinical practice preferences [17, 47]. This emphasizes the need for shared decision-making between patients and providers for RAD management, specifically regarding surgical interventions, and also highlights a potential area for further long-term prospective studies.

## Conclusion

RAD is a cause of disability and joint degeneration. Even with the successful identification and treatment of DDH in young children, RAD may be present in many patients with DDH. If left untreated, RAD can lead to significant long-term hip degeneration. The presentation of RAD is highly age-dependent and frequently subtle, often with a normal physical exam. Standard AP pelvic radiographs remain the most reliable imaging modality for evaluating RAD, with AI above two standard deviations of age norms suggesting the need for treatment. Treatment options consist of observation, abduction bracing, and pelvic osteotomy (with or without femoral osteotomy), and are generally guided by patient age, trends over time, and severity. Both abduction bracing and surgery have demonstrated the ability to significantly improve AI. There remains a crucial need for additional long-term, robust prospective studies to further evaluate the optimal treatment timeline and surgical indications.

##  Key References

- Shaw BA, Segal LS, SECTION ON ORTHOPAEDICS, et al. Evaluation and Referral for Developmental Dysplasia of the Hip in Infants.*Pediatrics* 2016; 138: e20163107.
  - ◌ Important reference demonstrating the epidemiology and clinical relevance of DDH and consequent RAD even when initially treated.
- Noordin S, Umer M, Hafeez K, et al. Developmental dysplasia of the hip.*Orthop Rev (Pavia)* 2010; 2: e19.
  - ◌ Highly cited reference that details the diagnosis and assessment of RAD in patients over the age of 6 months and the most relevant radiographic parameters.
- Gans I, Flynn JM, Sankar WN. Abduction Bracing for Residual Acetabular Dysplasia in Infantile DDH. *Journal of Pediatric Orthopaedics* 2013; 33: 714.
  - ◌ A recently published study documenting the significant improvement in AI with abduction bracing in patients with RAD compared to only observation.
- Swarup I, Talwar D, Sankar WN. Part-time Abduction Bracing in Infants With Residual Acetabular Dysplasia: Does Compliance Monitoring Support a Dose-dependent Relationship? *Journal of Pediatric Orthopaedics* 2021; 41: e125.
  - ◌ A recently published study demonstrating a dose-dependency of bracing time with improvement of AI in patients with RAD.
- Sawamura K, Kitoh H, Kaneko H, et al. Appropriate Surgical Timing of Salter Innominate Osteotomy for Residual Acetabular Dysplasia in Children.*J Pediatr Orthop* 2022; 42: e971–e975.
  - ◌ This recent study demonstrated that 90% of patients who received a Salter innominate osteotomy had satisfactory acetabular coverage at skeletal maturity, with older patients (cut-off of 8.6 years old) having a higher chance of sub-optimal postoperative remodeling.
- Tejpal T, Shanmugaraj A, Gupta A, et al. Outcomes and complications of patients undergoing Salter’s innominate osteotomies for hip dysplasia: a systematic review of comparative studies. *J Hip Preserv Surg* 2020; 7: 621–630.
  - ◌ A recent systematic review that robustly assesses and summarizes the treatment complications of patients undergoing pelvic osteotomy for hip dysplasia, serving as crucial data for providers to balance when managing RAD.

## Data Availability

No datasets were generated or analysed during the current study.
